# Safety of abatacept compared with other biologic and conventional synthetic disease-modifying antirheumatic drugs in patients with rheumatoid arthritis: data from an observational study

**DOI:** 10.1186/s13075-019-1921-z

**Published:** 2019-06-07

**Authors:** Gulsen Ozen, Sofia Pedro, Rebecca Schumacher, Teresa A. Simon, Kaleb Michaud

**Affiliations:** 1 University of Nebraska Medical Center, Omaha, NE USA; 2 FORWARD, The National Databank for Rheumatic Diseases, Wichita, KS USA; 3 Bristol-Myers Squibb, Princeton, NJ USA

**Keywords:** Abatacept, Autoimmune disease, Infection, Malignancy, Observational, Rheumatoid arthritis, Safety

## Abstract

**Background:**

To assess the risks of malignancies, infections and autoimmune diseases in patients with rheumatoid arthritis (RA) treated with abatacept compared with other biologic (b) disease-modifying antirheumatic drugs (DMARDs) or conventional synthetic (cs)DMARDs, in a US-wide observational RA cohort

**Methods:**

Data were reviewed from patients (≥ 18 years) with RA who were registered with FORWARD, the National Databank for Rheumatic Diseases, and who initiated abatacept, other bDMARDs or csDMARDs between 2005 and 2015. Patients who switched treatment during the study could be allocated to more than one group. The incidence rates (IRs) by treatment were calculated for malignancies, hospitalized infections and autoimmune diseases identified by six monthly questionnaires and medical records. The hazard ratios (HRs) (95% confidence intervals [CIs]) for all outcomes with abatacept compared with other bDMARDs or csDMARDs were determined using marginal structural models adjusted for clinical confounders.

**Results:**

In the study sample, 1496 initiated abatacept, 3490 initiated another bDMARD and 1520 initiated a csDMARD. The risk of malignancies with abatacept was not statistically significant versus other bDMARDs (HR [95% CI)] 1.89 [0.93, 3.84]) or versus csDMARDs (HR [95% CI] 0.93 [0.20, 4.27]). Patients receiving abatacept versus other bDMARDs were at a lower risk of hospitalized infections (HR [95% CI] 0.37 [0.18, 0.75]); the risk versus csDMARDs was lower with wide CIs (HR [95% CI] 0.31 [0.09, 1.05]). The relative risks for psoriasis were similar between treatment groups (HR [95% CI] 1.46 [0.76, 2.81] and HR [95% CI] 2.05 [0.59, 7.16] for abatacept versus other bDMARDs and versus csDMARDS, respectively). The IR (95% CI) of severe infusion/injection reactions was lower with abatacept compared with other bDMARDs (1.57 [1.11, 2.17] vs 2.31 [1.87, 2.82] per 100 patient-years, respectively).

**Conclusions:**

In this analysis, abatacept was well tolerated and did not result in an overall increased risk of malignancies, infections or autoimmune diseases compared with other bDMARDs or csDMARDs.

**Electronic supplementary material:**

The online version of this article (10.1186/s13075-019-1921-z) contains supplementary material, which is available to authorized users.

## Background

The introduction of biologic (b) disease-modifying antirheumatic drugs (DMARDs) into clinical practice has been a major advancement for patients with rheumatoid arthritis (RA) in whom treatment with conventional synthetic (cs)DMARDs has failed, and has contributed to the improved outcomes in RA reported over the previous two decades [1, 2]. The short-term safety of bDMARDs has been documented in clinical trials; however, the long-term safety of bDMARDs is of clinical interest in the population of patients with RA [3]. In particular, the risk of malignancies and hospitalized infections in patients with RA is higher than in the general population [4, 5], and there is a need to differentiate between the long-term effects of RA and those associated with bDMARD treatment. Additionally, most safety data in the literature relate to tumour necrosis factor-α inhibitors (TNFis), and more studies are needed relating to the safety of other bDMARDs and targeted synthetic DMARDs [3].

Abatacept is a fully human, selective T cell co-stimulation modulator that was approved in the USA for the treatment of RA in 2005. The efficacy of abatacept in RA in both biologic-naïve patients and in those with previous bDMARD failure has been demonstrated in several randomized controlled trials (RCTs) [6–15]. The safety findings across these studies were consistent between trials, and integrated analyses reflecting the long-term safety of intravenous (IV) and subcutaneous (SC) abatacept reported similar and stable incidence rates (IRs) of serious infections (SIs), malignancies, autoimmune events and infusion reactions, with no new safety signals over time [6–12, 14–17]. However, the relevance of these findings to the longer-term use of abatacept in clinical practice may be limited by the stringent patient inclusion criteria, short follow-up periods and limited power restraints for the detection of adverse events (AEs) in RCTs. Data from observational studies can supplement RCT safety data to broaden the understanding of the risks associated with abatacept treatment over time in a typically heterogeneous, clinical RA population. To date, observational findings for the safety of abatacept are mostly derived from biologic- or abatacept-specific registries of administrative or pharmacy data. Analyses of these data have generated conflicting results, possibly due to considerable variations in factors such as study population, comparators, outcome definitions, confounders and AEs [18–25]. Comparative observational data on the IRs of malignancies with abatacept are also inconsistent [26, 27]; however, when compared with other bDMARDs, abatacept has been associated with a higher relative risk of non-melanoma skin cancers (NMSCs) [23, 28]. Comparative data for the risk of SIs with abatacept versus other bDMARDs are similarly conflicting [19, 20, 22, 25, 29], and there are no data directly comparing the risk of SIs with abatacept versus csDMARDs [19, 25].

The abatacept global post-marketing epidemiology programme was designed to evaluate infection and malignancy risks associated with abatacept (initially intended as a first-line DMARD) compared with those of csDMARDs for the treatment of RA. The programme spanned more than 10 years and consists of observational studies based on biologic disease registries and healthcare claims databases, such as FORWARD (the National Databank for Rheumatic Diseases)—a US-wide registry that enrolls patients with rheumatic diseases in the community [29]. Information from patients is obtained from questionnaires every 6 months and is validated when required using medical records.

The aim of this analysis was to assess the risks of malignancies, infections, autoimmune diseases and mortality in patients with RA treated with abatacept compared with other bDMARDs or csDMARDs in an observational cohort of patients enrolled in the US FORWARD registry.

## Methods

This analysis utilized data from FORWARD, an ongoing, longitudinal, prospective, observational study in the USA, which has been described in detail previously [29]. Patient-recorded details collected by bi-annual questionnaires were analysed and included all medications taken in the previous 6 months (including doses, months taken, start and stop dates, reasons for discontinuation and side effects). Other information collected included patient demographics, socioeconomic data, co-morbidities, medical events, health-related quality of life, health symptoms and RA-specific outcome measures. The last questionnaire used for this analysis was administrated between January and June 2016 and reflected the events of the preceding 6-month period (August to December 2015) [29, 30]. The analysis population comprised all those who completed at least one full questionnaire and initiated a new course (incident users) of either abatacept, other bDMARDs (adalimumab, anakinra, certolizumab, etanercept, golimumab, infliximab, rituximab and tocilizumab) or csDMARDs (methotrexate, hydroxychloroquine, leflunomide and sulfasalazine) during the study period. The design of the analysis and inclusion of comparison arms helped mitigate complications with data capture. Each initiator of abatacept was matched with initiators of other RA treatments by date of cohort entry in a maximum of a 1:3 ratio. Patients in the abatacept group were defined as those who initiated abatacept or switched to abatacept after starting another bDMARD or a csDMARD. Patients in the comparator treatment groups were defined as those with no history of abatacept treatment. Patients in the comparator groups who switched to abatacept following treatment with another bDMARD or a csDMARD were included in the abatacept group and contributed to both the comparator (up to point of switch) and abatacept (following switch) data sets. Baseline was defined as the treatment start date; patients who switched treatment during the study could be allocated to more than one group. The database was locked on 4 June 2016.

### Patients

Patients enrolled in FORWARD and eligible for inclusion in this analysis were aged ≥ 18 years, had a confirmed diagnosis of RA, and initiated abatacept, another bDMARD or csDMARD between 1 July 2005 and 31 December 2015 in the USA. For each endpoint, except hospitalized infections, patients with the endpoint of interest at baseline were excluded from the analysis. For the analysis of malignancies, patients with a prior history of cancer at baseline were excluded. All patients provided their written informed consent for participation in the analysis.

### Analysis outcomes

The primary outcomes were malignancies (overall malignancies [including NMSC], lung cancer, lymphoma, breast cancer and NMSC), infections (hospitalized infections, pneumonia, opportunistic infections and tuberculosis) and autoimmune diseases (lupus, multiple sclerosis and psoriasis). Malignancies, infections and autoimmune diseases were identified from study questionnaires and were validated by patient and/or physician interview and medical record review, using the International Classification of Diseases, Ninth Revision, Clinical Modification diagnosis codes and hospitalization codes (see Additional file 1: Tables S1, Additional file 2: Tables S2, Additional file 3: Tables S3). Secondary outcomes included hospitalizations for any reason, any or serious infusion and/or injection reaction and death (identified and validated using the National Center for Health Statistics National Death Index). Reactions to infusions and injections were patient-reported only and were classified as ‘severe’ if they caused severe redness and/or pain, changes in blood pressure, difficulty breathing, feeling ill, chills, feeling faint or any severe symptoms that required medical care.

### Follow-up

Study follow-up started from initiation of abatacept, other bDMARDs or csDMARDs and continued until the first development of any study outcome, treatment discontinuation, death, loss to follow-up or end of study period, whichever came first. Hospitalized infections were attributed to the corresponding treatment group when the treatment was ongoing or discontinued ≤ 3 months previously. This risk window was extended to ≤ 12 months for rituximab due to its long-term effects on B cells. For the assessment of hospitalized infections, this risk window after treatment discontinuation was included in the follow-up period for patients who discontinued therapy for any other reason.

### Statistical analysis

Overall IRs for each outcome with 95% confidence intervals (CIs) were calculated by dividing the number of events for each endpoint by the total patient-time at risk. Only the occurrence of the first event of interest was considered, and the corresponding rates per 100 patient-years were determined. The risks of malignancies, infections and selected autoimmune disease infections were quantified in patients receiving abatacept versus other bDMARDs and abatacept versus csDMARDs using marginal structural models (MSMs). Results were presented as hazard ratios (HRs) with 95% CIs.

MSMs allow proper adjustment of time-varying confounders that are also affected by prior treatment [31]. For this analysis, the inverse probability of treatment weights (IPTWs) were derived from each patient’s treatment history and time-varying confounders. These IPTWs were then used in a logistic regression model to control for the time-dependent confounding in the outcome-treatment association, allowing less biased estimates to be obtained [32]. The IPTWs created a pseudo-population in which patients receiving treatment and those not receiving treatment were balanced over the time-varying confounders, but the relationship between treatment and outcome was not altered. The HR was obtained by using a weighted pooled logistic regression (which is equivalent to a Cox model when the hazard of treatment is small) for the probability of receiving abatacept at a given time using the following time-varying covariates measured at baseline and point of measurement: age, sex, employment status (yes/no), annual income (≤ 45 K USD, > 45 K USD), education level (≤ 12 years, 13–15 years, ≥ 16 years), smoking status (never, current and past), disease duration (≤ 3 years, 4–10 years, ≥ 11 years), Health Assessment Questionnaire-Disability Index (HAQ-DI), pain and patient global scores by visual analogue scale (0–10), body mass index, Rheumatic Diseases Comorbidity Index score, any chronic lung disease (yes/no), diabetes (yes/no), number of prior bDMARDs (0, 1, 2, ≥ 3), glucocorticoid (GC) use (yes/no, duration, daily dose [< 7.5 mg/day, 7.5–< 15 mg/day, ≥ 15 mg/day]), year of study entry (2005–2007, 2008–2010, 2011–2013, 2014–2015) and follow-up time using a three-knot spline. Considering the effects of increasing age, co-morbidities, disease duration, and severity measures on the outcomes, time-varying age, disease duration, HAQ-DI, pain and patient global scores, Rheumatic Disease Comorbidity Index (RDCI) and GC treatment duration were also included in the model. The dataset was discretized into one observation per month, and the hazard of treatment in any month was considered small [33]. Due to the low number of events for subtypes of malignancies, infections and autoimmune diseases, MSMs were created only for overall malignancies, NMSC and hospitalized infections.

In order to prevent bias from removing observations due to missing data, all missing covariates of the completed questionnaires were replaced by using multiple imputation by chained equations to create multiple imputed datasets for analyses. Since the odds ratios from MSMs are equivalent to the HRs that would be obtained from the Cox models, the results were presented as HR (95% CI). An intention-to-treat analysis was performed for all outcomes except hospitalized infections where an on-treatment plus 90 days was preferred. The weights were corrected for loss of follow-up and, for infection outcomes, induced selection bias due to artificial censoring (i.e. treatment noncompliance). All *p* values were two-sided and were conducted at a significance level of 0.05. All statistical analyses were performed using Stata/MP version 14.2 (StataCorp, College Station, TX, USA).

## Results

The analysis included 1496 patients who initiated abatacept with 4896 patient-years of total follow-up and 2502 patient-years of abatacept exposure; 3490 patients who initiated another bDMARD with 11,777 patient-years of total follow-up (9658 patient-years of drug exposure); and 1520 patients who initiated a csDMARD with 4816 patient-years of total follow-up (4184 patient-years of drug exposure). Median follow-up was 2.5 years per patient (interquartile range [IQR] 1.0–4.5 years) for abatacept and bDMARDs and 2.0 years per patient (IQR 1.0–4.0 years) for csDMARDs.

Patient baseline characteristics by treatment group are presented in Table 1. At baseline, patients initiating abatacept versus other bDMARDs or csDMARDs tended to have higher HAQ-DI, pain and patient global scores, and greater proportions of patients had prior use of csDMARDs and concurrent use of a GC. Prior use of a bDMARD was more frequent in abatacept versus other bDMARD initiators (≥ 2 bDMARDs, 50.2% vs 27.8%, respectively), and most patients in both groups were on a concomitant csDMARD (73% and 76%, respectively).

**Table 1:** Baseline patient characteristics by treatment

Variables	Abatacept *n* = 1496	Other bDMARDs *n* = 3490	csDMARDs *n* = 1520
Age, years, mean (SD)	61.5 (12.8)	60.9 (12.9)	63.3 (12.9)
Female	85.5	83.6	80.9
Education, years, mean (SD)	13.8 (2.3)	13.9 (2.3)	13.6 (2.4)
White	94.1	93.5	93.0
Disease duration, years, mean (SD)	16.6 (12.2)	16.6 (12.1)	15.3 (13.8)
Rheumatic Disease Comorbidity Index score (0–9), mean (SD)	2.1 (1.7)	2.0 (1.6)	2.0 (1.6)
Body mass index, kg/m^2^, mean (SD)	29.0 (7.3)	28.8 (7.1)	29.1 (7.3)
History of smoking	42.9	45.2	48.2
Hypertension	38.5	38.1	39.0
Diabetes	12.2	11.9	13.7
History of cancer	26.5	25.7	28.1
HAQ-DI score (0–3), mean (SD)	1.3 (0.7)	1.2 (0.7)	1.1 (0.7)
Pain score (0–10), mean (SD)	5.6 (2.7)	4.9 (2.8)	4.5 (2.8)
Patient global score (0–10), mean (SD)	4.9 (2.5)	4.4 (2.5)	4.2 (2.5)
Number of prior csDMARDs, mean (SD)	2.9 (1.9)	2.7 (1.8)	2.2 (1.5)
Number of prior bDMARDs, mean (SD)	1.7 (1.2)	1.0 (1.1)	0.2 (0.6)
0	15.2	41.9	89.3
1	34.6	30.3	6.9
2–3	44.3	24.5	3.4
≥ 4	5.9	3.3	0.5
Concurrent methotrexate	51.7	55.0	45.7
Concurrent other non-MTX csDMARDs	21.0	21.0	37.0
Concurrent glucocorticoid^†^	43.0	34.0	31.0
< 7.5 mg/day	63.7	64.9	65.7
7.5–< 15 mg/day	25.6	25.1	22.2
≥ 15 mg/day	10.8	10.1	12.1
Concurrent NSAID	41.3	42.8	44.5

The values are presented as % unless indicated otherwise. Baseline is treatment start date; patients who switched treatment during the study could be allocated to more than one group

^†^As prednisone dose equivalents

*bDMARD* biologic disease-modifying antirheumatic drug, *csDMARD* conventional synthetic disease-modifying antirheumatic drug, *HAQ-DI* Health Assessment Questionnaire-Disability Index, *MTX* methotrexate, *NSAID* nonsteroidal anti-inflammatory drug, *SD* standard deviation

### Outcomes

**Fig. 1:**
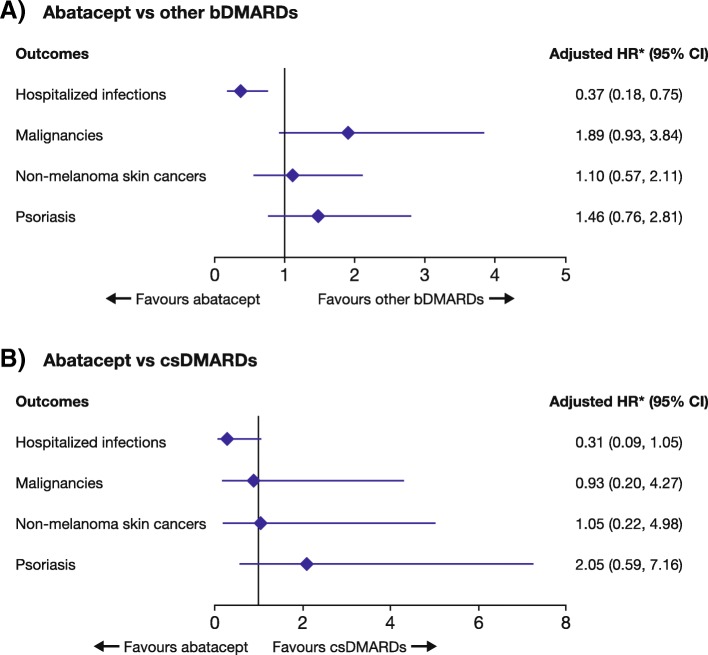
Association of treatment with hospitalized infections, malignancies and psoriasis in patients with RA. **a** Abatacept vs other bDMARDs. **b** Abatacept vs other csDMARDs. *Using inverse probability of treatment weights with further adjustment for time-varying age, disease duration, HAQ-DI, pain and patient global scores, RDCI, and GC treatment duration. bDMARD = biologic disease-modifying antirheumatic drug; CI = confidence interval; csDMARD = conventional synthetic disease-modifying antirheumatic drug; GC = glucocorticoid; HAQ-DI=Health Assessment Questionnaire-Disability Index; HR = hazard ratio; RA = rheumatoid arthritis; RCDI = Rheumatic Disease Comorbidity Index

In the fully adjusted MSMs, there were no differences in the risks of malignancies with abatacept relative to other bDMARDs or to csDMARDs (Fig. 1). The relative risks of breast cancer, lung cancer and lymphoma could not be determined because none of these events was observed in any treatment group. Overall, abatacept treatment was associated with a lower risk of all hospitalized infections compared with other bDMARDs (adjusted HR [95% CI] 0.37 [0.18, 0.75]), and with a lower risk (wide CIs) compared with csDMARDs (0.31 [0.09, 1.05]). When analysis was limited to line of therapy (second-line, third-line or greater), the risk difference in hospitalized infections between abatacept and other bDMARDs and csDMARDs decreased (Table 2). The number of overall autoimmune events was low, and most models used to analyse the relative risks of these secondary outcomes did not converge. There was a numerical increase in the risk of psoriasis with abatacept compared with other bDMARDs and with csDMARDs. The relative risks of hospitalized infections, malignancies and NMSCs by abatacept treatment line are presented in Table 2.

**Table 2:** Risk of hospitalized infections and malignancies associated with abatacept in RA by line of treatment

Outcomes	Using inverse probability of treatment weights					
	Abatacept vs other bDMARDs			Abatacept vs csDMARDs		
	No. of cases/no. of patients*	Unadjusted HR (95% CI)	Adjusted HR^†^ (95% CI)	No. of cases/no. of patients*	Unadjusted HR (95% CI)	Adjusted HR^†^ (95% CI)
Hospitalized infections						
Overall	37/1099 vs 25/3138	0.39 (0.17, 0.87)	0.37 (0.18, 0.75)	37/1099 vs 20/1103	0.52 (0.20, 1.39)	0.31 (0.09, 1.05)
First-line	1/171 vs 13/1191	NR	NR	1/171 vs 15/304	NR	NR
Second-line	15/409 vs 7/1132	0.49 (0.23, 1.03)	0.42 (0.18, 0.97)	15/409 vs 4/119	0.99 (0.26, 3.76)	NR
Third- or greater line	21/659 vs 5/1192	0.76 (0.42, 1.38)	0.64 (0.35, 1.15)	21/659 vs 1/50	0.47 (0.13, 1.68)	0.42 (0.09, 2.10)
Malignancies*						
Overall	22/1099 vs 27/3138	2.32 (0.84, 6.44)	1.89 (0.93, 3.84)	22/1099 vs 24/1103	0.77 (0.29, 2.06)	0.93 (0.20, 4.27)
First-line	3/171 vs 14/1191	NR	NR	3/171 vs 17/304	0.75 (0.09, 6.13)	0.36 (0.03, 3.83)
Second-line	6/409 vs 8/1132	4.61 (0.98, 21.7)	NR	6/409 vs 6/119	NR	NR
Third- or greater line	13/659 vs 5/1192	0.35 (0.04, 3.08)	1.34 (0.39, 4.57)	13/659 vs 1/50	0.61 (0.06, 5.75)	NR
Non-melanoma skin cancers						
Overall	37/1099 vs 25/3138	1.26 (0.69, 2.29)	1.10 (0.57, 2.11)	37/1099 vs 20/1103	0.90 (0.33, 2.46)	1.05 (0.22, 4.98)
First-line	1/171 vs 13/1191	NR	NR	1/171 vs 15/304	0.88 (0.10, 7.37)	0.62 (0.05, 7.28)
Second-line	15/409 vs 7/1132	4.08 (0.80, 20.67)	NR	15/409 vs 4/119	NR	NR
Third- or greater line	21/659 vs 5/1192	0.35 (0.0, 3.08)	1.34 (0.39, 4.57)	21/659 vs 1/50	0.61 (0.06, 5.75)	NR

*Patients with prior history of malignancy were excluded

^†^Using inverse probability of treatment weights with further adjustment for time-varying age and disease duration, HAQ-DI, pain and patient global scores, RDCI and GC treatment duration

*bDMARD* biologic synthetic disease-modifying antirheumatic drug, *CI* confidence interval, *csDMARD* conventional synthetic disease-modifying antirheumatic drug, *GC* glucocorticoid, *HAQ-DI* Health Assessment Questionnaire-Disability Index, *HR* hazard ratio, *NR* not reported due to low event numbers causing inability to achieve convergence, *RA* rheumatoid arthritis, *RCDI* Rheumatic Disease Comorbidity Index

For the secondary outcomes (except psoriasis), the results were presented as crude IRs due to the low number of events and inability to achieve convergence by the MSMs.

The IR of overall malignancy was higher in the abatacept group than in the other bDMARD group, and was lower than in the csDMARD group (Table 3). The most frequently observed malignancy was NMSC in all treatment groups.

**Table 3:** Incidence rates for malignancy, infections and autoimmune disease outcomes by treatment

Outcomes	Abatacept		Other bDMARDs		csDMARDs	
	No. of events/ no. of patients	IR (95% CI)*	No. of events/ no. of patients	IR (95% CI)*	No. of events/ no. of patients	IR (95% CI)*
Overall malignancy^†^	22/1099	0.76 (0.47, 1.15)	27/2592	0.50 (0.33, 0.72)	24/1093	0.86 (0.55, 1.27)
Lung	1/1099	0.012 (0.00, 0.07)	1/2592	0.02 (0.00, 0.10)	0/1093	0.00 (0.00, 0.13)
NMSC	20/1099	0.55 (0.40, 0.73)	25/2592	0.46 (0.30, 0.68)	20/1093	0.71 (0.43, 1.10)
Hospitalized infections	37/1496	1.63 (1.15, 2.24)	135/3490	1.78 (1.49, 2.11)	77/1520	1.90 (1.50, 2.38)
Opportunistic	2/1496	0.09 (0.01, 0.31)	6/3490	0.08 (0.03, 0.17)	3/1520	0.07 (0.02, 0.21)
Pneumonia	13/1496	0.57 (0.30, 0.97)	80/3490	1.04 (0.82, 1.29)	50/1520	1.03 (0.79, 1.33)
Lupus	1/1496	0.04 (0.00, 0.24)	0/3490	0.00 (0.00, 0.05)	3/1520	0.07 (0.02, 0.21)
Psoriasis	15/1496	0.66 (0.37, 1.09)	30/3490	0.39 (0.26, 0.55)	15/1520	0.36 (0.20, 0.59)
Multiple sclerosis^†^	1/1484	0.04 (0.00, 0.24)	0/3471	0.00 (0.00, 0.05)	0/1515	0.00 (0.00, 0.09)
All hospitalizations	637/1496	28.83 (26.64, 31.16)	2001/3490	28.26 (27.03, 29.53)	1292/1520	32.53 (30.78, 34.36)
All deaths^‡^	128/1496	2.99 (2.49, 3.55)	162/3490	2.16 (1.84, 2.52)	117/1520	2.78 (2.30, 3.33)

There were no events for breast cancer, lymphoma or tuberculosis in any treatment group

*Per 100 patient-years

^†^Patients with prior history of malignancy or multiple sclerosis were excluded

^‡^Deaths from all causes within 1 year of last use of abatacept or of last study observation for control subjects

*bDMARD* biologic disease-modifying antirheumatic drug, *CI* confidence interval, *csDMARD* conventional synthetic disease-modifying antirheumatic drug, *IR* incidence rate, *NMSC* non-melanoma skin cancer

The IRs of infections were similar across treatment groups, with lower rates of hospitalized infections or pneumonia with abatacept versus other bDMARDs or csDMARDs (Table 3). No cases of tuberculosis were observed in any treatment group. The IRs of autoimmune diseases were mostly similar across treatments; however, a higher incidence of psoriasis was reported in the abatacept versus other treatment groups (Table 3). The highest frequency of hospitalizations was observed in the csDMARD treatment group, and death rates were comparable across treatments (Table 3).

The IRs for any reaction to treatment infusion or injection were lower with abatacept than with other bDMARDs; severe administration reactions were less common with SC than with IV abatacept (Table 4).

**Table 4:** Incidence rates of infusion/injection reactions by bDMARD treatment

Outcomes	Abatacept		Other bDMARDs					
			Overall		TNFi		Non-TNFi bDMARDs	
	No. of events/ no. of patients	IR (95% CI)*	No. of events/ no. of patients	IR (95% CI)*	No. of events/ no. of patients	IR (95% CI)*	No. of events/ no. of patients	IR (95% CI)*
Any infusion reaction	115/1330	5.36 (4.42, 6.43)	196/1336	9.25 (8.00, 10.64)	106/985	7.00 (5.73, 8.47)	74/501	9.38 (7.36, 11.77)
Severe infusion reactions	27/1330	1.26 (0.83, 1.83)	41/1336	1.94 (1.39, 2.63)	24/985	1.59 (1.02, 2.36)	12/501	1.52 (0.79, 2.66)
Any injection reaction	53/626	8.19 (6.13, 10.71)	573/1690	23.43 (21.55, 25.43)	550/1679	22.62 (20.76, 24.59)	3/20	12.46 (2.57, 36.41)
Severe injection reactions	3/626	0.46 (0.10, 1.35)	46/1690	1.88 (1.38, 2.51)	40/1679	1.65 (1.18, 2.24)	1/20	4.15 (0.11, 23.14)
Severe injection or infusion reactions	37/1496	1.57 (1.11, 2.17)	95/2648	2.31 (1.87, 2.82)	65/2361	1.85 (1.42, 2.35)	14/512	1.75 (0.95, 2.93)

*Per 100 patient-years

*bDMARD* biologic disease-modifying antirheumatic drug, *CI* confidence interval, *IR* incidence rate, *TNFi* tumour necrosis factor-α inhibitor

## Discussion

In this US-wide observational cohort study of patients with RA, abatacept was associated with low IRs of malignancies, infections, autoimmune diseases, infusion/injection reactions and mortality, with no new safety signals. The incidence of infusion/injection-site reactions was lower with abatacept than with other bDMARDs. The risks of overall malignancies, infections and autoimmune diseases were comparable across treatment groups; however, the risk of hospitalized infection was lower with abatacept than with other bDMARDs.

Our study showed a low IR of new malignancies with abatacept. To date, only two observational studies (Sweden national cohort and CORRONA registry) have examined the comparative risk of malignancy associated with abatacept; they reported an increased risk of NMSC with abatacept compared with csDMARDs (HR [95% CI] 2.15 [1.31, 3.52]) [23] and methotrexate (15.3 [2.05, 114.0]) [28], whereas the comparative risks of haematological and solid malignancies were similar [23, 28]. In contrast to our analysis, both studies included patients with a prior history of malignancy, which was reflected in the higher rates of malignancy observed. The power restraints and inadequate follow-up duration of RCTs for the detection of malignancies and the scarcity of observational data suggest further research is warranted to improve understanding of the risk of malignancy associated with abatacept, particularly in comparison with other bDMARDs.

The incidence of hospitalized infections with abatacept in this analysis was lower than that reported in other observational studies, possibly due to differences in the characteristics of the study populations, such as patients’ sex and co-morbidities [20, 25], or in the definitions used for infections [22].

Following adjustment for clinical factors influencing infection risk, we found that the overall risk of hospitalized infections with abatacept was lower than that of other bDMARDs and csDMARDs. When limited by line of therapy, the risk difference in hospitalized infections between abatacept and other bDMARD groups decreased, possibly due to infections from prior drugs precluding further use of bDMARDs. Although the comparative data currently available are limited and inconsistent, our findings are consistent with an overall trend toward a lower risk of hospitalized infections or SIs with abatacept compared with other bDMARDs [19, 22, 25, 34]. Observational findings from administrative datasets have shown that compared with other bDMARDs, abatacept was associated with a lower risk of SIs (19, 25). Conversely, other observational studies found that SI risk with abatacept was comparable with that of rituximab [22] and etanercept [34]. Although the risk of SIs with abatacept compared with other bDMARDs observed in our analysis was lower, the risk estimate was smaller. This difference could be due to variables such as the mean age of the study population, treatment episodes (incident/prevalent use), treatment line, comparator groups, covariates included and methods used to address channelling bias. The hospitalized infection risk with abatacept treatment compared with csDMARDs in the present study is consistent with an epidemiologic assessment of data from seven clinical trials that showed the IRs of SIs with abatacept were comparable with those in patients with RA treated with csDMARDs [35]. However, we observed a lower hospitalized infection risk with abatacept treatment compared with csDMARDs despite being not significant. At baseline, csDMARD initiators were slightly older, had higher smoking frequencies and had a slightly higher dose of glucocorticoid use; these are potentially associated with infection risk. Although we used an appropriate methodology, MSM, to address the channelling bias, as an observational study there may be residual channelling due to unmeasured confounders.

In this analysis, the IRs of most autoimmune diseases were similar across treatment groups. Psoriasis was the most frequently occurring autoimmune disease in all treatment groups; however, the IRs were within the ranges reported elsewhere [13, 17]. Patients with a pre-existing autoimmune disease are known to be at an increased risk of developing another [36–38]. Psoriasis, a T cell-mediated autoimmune disease, is known to coexist with RA more frequently than with osteoarthritis [38]. As expected, we did not observe an altered risk of psoriasis with abatacept treatment compared with other bDMARDs and csDMARDs.

We observed low IRs of infusion/injection reactions with abatacept treatment. In this analysis, use of IV abatacept was associated with a slightly higher IR of severe reactions than the SC formulation, possibly due to the chronological availability of the two formulations, leading to the inclusion of fewer patients treated with SC abatacept and correspondingly shorter follow-up periods than for those treated with the IV formulation. A phase IIIb noninferiority study to compare the efficacy and safety of SC versus IV abatacept reported similar incidences of infusion and injection reactions (2.2% vs 2.7%, respectively) [39]. In this analysis, lower IRs of infusion/injection reactions were observed with abatacept versus TNFis and other bDMARDs, which is consistent with the ATTEST and AMPLE trials that showed infusion and injection reactions were more frequent with infliximab and adalimumab than with abatacept [11, 16]. Furthermore, an examination of healthcare data indicated the IRs of hypersensitivity reactions were lower for abatacept than for other injectable bDMARDs [24].

Although early, aggressive treatment strategies and the use of bDMARDs have improved treatment outcomes in RA [1, 40], safety concerns have been raised [3]. Research on the comparative risks and benefits of treatment using observational data have become critical, as comparative safety data from RCTs are scarce. Conducting an appropriate analysis to address channelling bias in observational studies is challenging; however, the use of MSM methodology to adjust for time-varying confounders by weighting for the treatment groups where the baseline characteristics were balanced allowed us to obtain less biased estimates [32]. This methodology also addresses the attrition bias between treatment groups by weighting for the censoring distribution and balancing the characteristics of patients lost to follow-up with those who were followed up.

Despite using a strong methodology, our study had some limitations. First, the numbers of events, and accordingly incidence rates, reported were lower than in previous observational studies, which precluded the construction of models to assess the risk of different malignancies and hospitalized infections. It is possible that the inclusion of different study populations and the application of the strict validation process in FORWARD, although increasing the accuracy of the events reported, may be among the reasons for lower incidence rates of safety events than in previous observational studies. In addition, patients who are possibly in better health may be more likely to participate in FORWARD than those who are frail and at higher risk of infection and cancer. This participation bias can also explain the low incidence rates of the events. Moreover, due to the self-reported nature of the data, some events may not have been recorded. Second, our sample size was inadequate to examine the safety risks associated with abatacept compared with individual bDMARDs or classes of bDMARDs. Furthermore, the analysis population included both biologic-naïve and biologic-experienced patients, for whom safety risks could differ. In order to address this, we added the number of previous bDMARDs to the model. We also performed a subgroup analysis on biologic-naïve and biologic-experienced patients (Table 2); however, the sample size was not adequate to see trends in safety risks. There is the potential for time-lag bias due to the 10-year follow-up period, particularly as patients were allowed to contribute to different treatment groups and drug indications may change over time. However, calendar matching will have minimized this potential bias [41]. The matching did not take into consideration that patients may switch treatments; however, continuing to include switchers in the study increased the number of patients included. Lastly, although our analyses included several clinical confounders including disease severity measures, unmeasured confounders could be present in this observational study.

## Conclusions

Our study showed that abatacept treatment in patients with RA is associated with low IRs of malignancies, infections, autoimmune diseases and infusion/injection reactions. Our findings suggest the risks of hospitalized infections, malignancies and psoriasis are similar with abatacept and csDMARDs and that the safety profile for abatacept versus other bDMARDs is favourable in terms of hospitalized infections and infusion/injection reactions. Given that patients with RA are already at a higher risk of malignancy and infections than the general population, our findings are likely to be of clinical interest in making therapeutic decisions. However, additional observational research with longer follow-up periods is needed to fully evaluate the comparative safety of abatacept versus other DMARDs in terms of malignancy and mortality.

## Additional files

Additional file 1: **Table S1.** ICD-9-CM codes for hospitalized infections. (DOCX 12 kb)

Additional file 2: **Table S2.** ICD-9-CM codes for malignancies. (DOCX 12 kb)

Additional file 3: **Table S3.** ICD-9-CM codes for autoimmune diseases. (DOCX 11 kb)

## Data Availability

Bristol-Myers Squibb policy on data sharing may be found at https://www.bms.com/researchers-and-partners/independent-research/data-sharing-request-process.html.

## Abbreviations

AEs: Adverse events
bDMARDs: Biologic DMARDs
CIs: Confidence intervals
csDMARDs: Conventional synthetic DMARDs
DMARDs: Disease-modifying antirheumatic drugs
GC: Glucocorticoid
HAQ-DI: Health Assessment Questionnaire-Disability Index
HR: Hazard ratios
IPTWs: Inverse probability of treatment weights
IRs: Incidence rates
IV: Intravenous
MSMs: Marginal structural models
NMSCs: Non-melanoma skin cancers
RA: Rheumatoid arthritis
RCTs: Randomized controlled trials
SIs: Serious infections
TNFis: Tumour necrosis factor-α inhibitors
