# Supplementary material for: Safety of abatacept compared with other biologic and conventional synthetic disease-modifying antirheumatic drugs in patients with rheumatoid arthritis: data from an observational study
Source: Arthritis Res Ther. 2019 Jun 7;21:141. doi: 10.1186/s13075-019-1921-z (PMC6555014; doi:10.1186/s13075-019-1921-z)
Supplement: Supplementary file 1 — Table S1. ICD-9-CM codes for hospitalized infections. (DOCX 12 kb) [file 13075_2019_1921_MOESM1_ESM.docx]

Table S1. ICD-9-CM codes for hospitalized infections

| Outcome: Hospitalized infections | ICD-9-CM code |
| --- | --- |
| Tuberculosis | 010/011.39, 011.5/018.99 |
| Atypical mycobacterial infections | 031* |
| Cryptococcosis | 117.5 |
| Histoplasmosis | 115.0/115.99 |
| Aspergillosis | 117.3 |
| Coccidiodomycosis | 114.0/114.99 |
| Cryptococcosis | 117.5 |
| Blastomycosis | 116/116.0 |
| Candidiasis | 112.5 |
| Cytomegalovirus | 078.7 484.1 |
| Viral Hepatitis | 070/070.9 |
| Hepatitis B | 070.2/070.33 |
| Hepatitis C | 070.41, 070.44, 070.51, 070.54, 070.7/070.71 |
| Cryptosporidiosis | 007.4 |
| Toxoplasmosis | 130/130.9 |
| Pneumocystis carinii | 136.3 |
| Human papillomavirus | 079.4 |
| Herpes simplex | 054/054.9 |
| Herpes zoster | 053/053.9 |
| Listeriosis | 010/011.39, 011.5/018.99, 027.0, 117.3, 115.0/115.99, 117.5, 136.3, 112.5,  078.7, 484.1, 114.0/114.99, 054.3, 117.7, 116/116.0, 031* |
| Pneumonia | 480/487.0, 513/513.0, 003.22 |
| Sepsis/bacteremia | 038*, 995.91, 790.7 |
| Cellulitis | 681/682.9 |
| Meningitis/encephalitis | 320/320.9, 036/036.2, 323/323.9, 049.2, 091.81, 054.3, 062/063.9, 066.4/066.49, 094.81, 094.2, 094.81 |
| Endocarditis | 421/421.9, 422.92, 391.2, 036.42, 093.2/093.29, 098.84 |
| Pyelonephritis | 590/590.99 |
| Septic arthritis | 711.0/711.09, 711.9/711.99, 003.23, 098.5/098.59 |
| Osteomyelitis | 730/730.29, 003.24, 376.03 |
| Prosthetic joint infection | 996.66 |
| Skin | 684/686.9 |
| Other | 009* |

*Includes all codes within this diagnosis code

ICD-9-CM=International Classification of Diseases, Ninth Revision, Clinical Modification
