# Supplementary material for: Safety of abatacept compared with other biologic and conventional synthetic disease-modifying antirheumatic drugs in patients with rheumatoid arthritis: data from an observational study
Source: Arthritis Res Ther. 2019 Jun 7;21:141. doi: 10.1186/s13075-019-1921-z (PMC6555014; doi:10.1186/s13075-019-1921-z)
Supplement: Supplementary file 2 — Table S2. ICD-9-CM codes for malignancies. (DOCX 12 kb) [file 13075_2019_1921_MOESM2_ESM.docx]

Table S2. ICD-9-CM codes for malignancies

| Outcome: Malignancy | ICD-9-CM code |
| --- | --- |
| Bladder | 188* |
| Bone | 170* |
| Brain | 191* |
| Breast | 174* 175* |
| Cervix | 180* |
| Colon | 153* 154* |
| Endocrine | 193* 194* |
| Esophagus | 150* |
| Eye | 190* |
| Gall bladder | 156* |
| Hodgkin’s Disease | 201* |
| Head and neck | 141*, 145*, 146*, 148*, 160*, 161* |
| Kidney | 189* |
| Leukemia | 203/207.8 |
| Liver | 155* |
| Lung | 162*, 163* |
| Malignant tumours of lymphatic/lymphoid tissue | 200/202.9 |
| Malignant melanoma | 172* |
| Non-Hodgkin lymphoma | 200*, 202* |
| Ovary | 183* |
| Pancreas | 157* |
| Penis | 187* |
| Peritoneum | 158* |
| Prostate | 185* |
| Non-Melanoma skin cancers | 173* |
| Soft tissue | 171* |
| Stomach | 151* |
| Testis | 186* |
| Uterus | 179* |
| Vagina | 184* |
| All malignancies | 140/208.9 |
| Solid malignancies | 140/199.9 |

*Includes all codes within this diagnosis code

ICD-9-CM=International Classification of Diseases, Ninth Revision, Clinical Modification
