# Supplementary material for: Safety of abatacept compared with other biologic and conventional synthetic disease-modifying antirheumatic drugs in patients with rheumatoid arthritis: data from an observational study
Source: Arthritis Res Ther. 2019 Jun 7;21:141. doi: 10.1186/s13075-019-1921-z (PMC6555014; doi:10.1186/s13075-019-1921-z)
Supplement: Supplementary file 3 — Table S3. ICD-9-CM codes for autoimmune diseases. (DOCX 11 kb) [file 13075_2019_1921_MOESM3_ESM.docx]

Table S3. ICD-9-CM codes for autoimmune diseases

| Outcome: Autoimmune diseases | ICD-9-CM code |
| --- | --- |
| Multiple sclerosis | 340 |
| Demyelinating diseases | 270.1, 277.86, 330.0, 341*, 357.81, 356.3 |
| Psoriasis | 696.1 |
| Lupus | 710.0 |

*****Includes all codes within this diagnosis code

ICD-9-CM=International Classification of Diseases, Ninth Revision, Clinical Modification
